# Seizures, cysticercosis and rural-to-urban migration: the PERU MIGRANT study

**DOI:** 10.1111/tmi.12456

**Published:** 2015-02-03

**Authors:** Isidro Gonzales, J Jaime Miranda, Silvia Rodriguez, Victor Vargas, Alfredo Cjuno, Liam Smeeth, Armando E Gonzalez, Victor C W Tsang, Robert H Gilman, Hector H Garcia

**Affiliations:** 1 Cysticercosis Unit, Instituto Nacional de Ciencias NeurologicasLima, Peru; 2 CRONICAS Centre of Excellence in Chronic Diseases, Universidad Peruana Cayetano HerediaLima, Peru; 3 Department of Medicine, School of Medicine, Universidad Peruana Cayetano HerediaLima, Peru; 4 Faculty of Epidemiology and Population Health, London School of Hygiene and Tropical MedicineLondon, UK; 5 School of Veterinary Medicine, Universidad Nacional Mayor de San MarcosLima, Peru; 6 Georgia State UniversityAtlanta, GA, USA; 7 Department of International Health, Johns Hopkins Bloomberg School of Public HealthBaltimore, MD, USA; 8 Department of Microbiology, School of Sciences, Universidad Peruana Cayetano HerediaLima, Peru; 9 Center for Global Health, Universidad Peruana Cayetano HerediaTumbes, Peru

**Keywords:** cysticercosis, neurocysticercosis, seizures, migration, *Taenia solium*, Peru

## Abstract

**Objectives:**

To examine the prevalence of seizures, epilepsy and seropositivity to cysticercosis in rural villagers (cysticercosis-endemic setting), rural-to-urban migrants into a non-endemic urban shanty town and urban inhabitants of the same non-endemic shanty town.

**Methods:**

Three Peruvian populations (*n* = 985) originally recruited into a study about chronic diseases and migration were studied. These groups included rural inhabitants from an endemic region (*n* = 200), long-term rural-to-urban migrants (*n* = 589) and individuals living in the same urban setting (*n* = 196). Seizure disorders were detected by a survey, and a neurologist examined positive respondents. Serum samples from 981/985 individuals were processed for cysticercosis antibodies on immunoblot.

**Results:**

Epilepsy prevalence (per 1000 people) was 15.3 in the urban group, 35.6 in migrants and 25 in rural inhabitants. A gradient in cysticercosis antibody seroprevalence was observed: urban 2%, migrant 13.5% and rural group 18% (*P* < 0.05). A similarly increasing pattern of higher seroprevalence was observed among migrants by age at migration. In rural villagers, there was strong evidence of an association between positive serology and having seizures (*P* = 0.011) but such an association was not observed in long-term migrants or in urban residents. In the entire study population, compared with seronegative participants, those with strong antibody reactions (≥ 4 antibody bands) were more likely to have epilepsy (*P* < 0.001).

**Conclusions:**

It is not only international migration that affects cysticercosis endemicity; internal migration can also affect patterns of endemicity within an endemic country. The neurological consequences of cysticercosis infection likely outlast the antibody response for years after rural-to-urban migration.

**Objectifs:**

Examiner la prévalence des crises d’épilepsie, de l’épilepsie et de la séropositivité à la cysticercose chez les villageois des zones rurales (cadre endémique pour la cysticercose), chez les migrants des zones ruraux vers les zones urbaines dans un bidonville urbain non endémique et chez les habitants urbains du même bidonville non endémique.

**Méthodes:**

Trois populations péruviennes (n = 985) recrutées initialement dans une étude sur les maladies chroniques et la migration, ont été étudiées. Ces groupes comprenaient des habitants de zones rurales d'une région d'endémie (n = 200), des migrants de long terme de zones ruraux vers les villes (n = 589) et les personnes vivant dans le même milieu urbain (n = 196). Les troubles épileptiques ont été détectés par un sondage et un neurologue a examiné les répondants positifs. Des échantillons de sérum de 981/985 individus ont été testés pour les anticorps de cysticercose par Immunoblot.

**Résultats:**

La prévalence de l’épilepsie (pour 1000 personnes) était de 15,3 dans le groupe urbain; 35,6 chez les migrants et 25 dans la population rurale. Un gradient dans la séroprévalence des anticorps de la cysticercose a été observé: dans le groupe urbain 2%, le groupe de migrants 13,5% et le groupe rural 18% (p <0,05). Une tendance croissante similaire de séroprévalence plus élevée a été observée chez les migrants selon l’âge à la migration. Chez les villageois ruraux, il y avait des preuves solides d'une association entre une sérologie positive et le fait d'avoir des crises (p = 0,011), mais une telle association n'a pas été observée chez les migrants de long terme ou chez les résidents urbains. Dans l'ensemble de la population de l’étude, ceux avec de fortes réactions d'anticorps (≥ 4 bandes d'anticorps) étaient plus susceptibles d'avoir l’épilepsie (p <0,001) comparé aux participants séronégatifs.

**Conclusions:**

La migration internationale n'est pas la seule qui affecte l'endémicité de la cysticercose; la migration interne peut aussi modifier les profils d'endémicité au sein d'un même pays d'endémie. Les conséquences neurologiques de l'infection par la cysticercose sont susceptibles de survivre à la réponse d'anticorps durant des années après la migration des zones rurales vers les zones urbaines.

**Objetivos:**

Examinar la prevalencia de convulsiones, epilepsia, y seropositividad para cisticercosis entre población rural (de zonas endémicas para cisticercosis), inmigrantes provenientes de zonas rurales a tugurios urbanos no endémicos, y habitantes urbanos de los mismo tugurios urbanos no endémicos.

**Métodos:**

Se estudiaron tres poblaciones peruanas (n=985) originalmente reclutadas en un estudio de enfermedades crónicas y migración. Estos grupos incluían habitantes rurales de una región endémica (n=200), inmigrantes de larga duración de zonas rurales a urbanas (n=589), e individuos que vivían en la misma zona urbana (n=196). Las convulsiones se detectaron mediante una encuesta y un neurólogo examinó a quienes habían respondido positivamente. Se procesaron muestras de suero de 981/985 individuos en busca de anticuerpos para cisticercosis mediante inmunoblot.

**Resultados:**

La prevalencia de epilepsia (por 1,000 personas) era de 15.3 en el grupo urbano, 35.6 en inmigrantes y 25 en habitantes rurales. Se observó un gradiente en la seroprevalencia de los anticuerpos para cisticercosis: grupos urbano 2%, inmigrante 13.5% y rural 18% (p<0.05). Se observó un patrón de aumento similar de mayor seroprevalencia entre inmigrantes según la edad que tenían en el momento de emigrar. En pobladores rurales, había una evidencia importante de asociación entre tener una serología positiva y sufrir convulsiones (p=0.011), pero esta asociación no se observaba en inmigrantes de larga duración o residentes urbanos. En la población al completo, comparada con los participantes seronegativos, aquellos con una fuerte reactividad de anticuerpos (≥4 bandas de anticuerpos) tenían una mayor probabilidad de sufrir epilepsia (p<0.001).

**Conclusiones:**

No solo la migración internacional afecta la endemicidad de cisticercosis; la migración interna también puede afectar los patrones de endemicidad dentro de un país endémico. Las consecuencias neurológicas de la infección por cisticercos podrían durar más que la respuesta a anticuerpos años después de la migración de zonas rurales a zonas urbanas.

## Introduction

Neurocysticercosis (NCC) is the most frequent helminthic infection of the human central nervous system, caused by the larvae of the pork tapeworm *Taenia solium*. A growing global public health problem 1, NCC is endemic in most developing countries, where it constitutes the main cause of secondary epilepsy. NCC is also emerging in industrialised countries because of migration from endemic zones 2.

Closely linked to poverty as for most zoonotic infections, the bulk of cysticercosis transmission occurs in rural villages where domestic pig raising coexists with poor sanitation and lack of sewage and potable water facilities. There are multiple reports of NCC in travellers and migrants to industrialised countries 2–7. Migration also occurs within countries, usually mobilising people from disease-endemic regions to more urban centres. However, data on the variations in prevalence and expression of NCC following migration within endemic regions is scarce or non-existent.

In the particular case of Peru, between the 1970s and the 1990s, prolonged terrorism-induced instability and insecurity (*Shining Path*) led thousands in rural areas to abandon their communities and move to large urban areas, primarily Lima, where they established shanty towns 8. Taking advantage of, and expanding upon a study on migration and chronic diseases already ongoing in Peru 9,10, we examined the prevalence of seizures, epilepsy and seropositivity to cysticercosis in three populations of individuals older than 30 years: rural villagers from a cysticercosis-endemic setting, long-term rural-to-urban migrants into a non-endemic urban shanty town and urban inhabitants born and living in the same non-endemic shanty town.

## Materials and methods

### Study design and study populations

This cross-sectional study assessed the prevalence of seizures and the prevalence of specific antibodies to cysticercosis in serum of three population-based groups: *Rural*, people born in Ayacucho who had always lived in a rural environment; *Rural-to-urban Migrants*, people born in Ayacucho who migrated from rural to urban areas and currently living in Lima, an 8-million metropolis; and *Urban*, people born and currently living in Lima.

**Figure 1:**
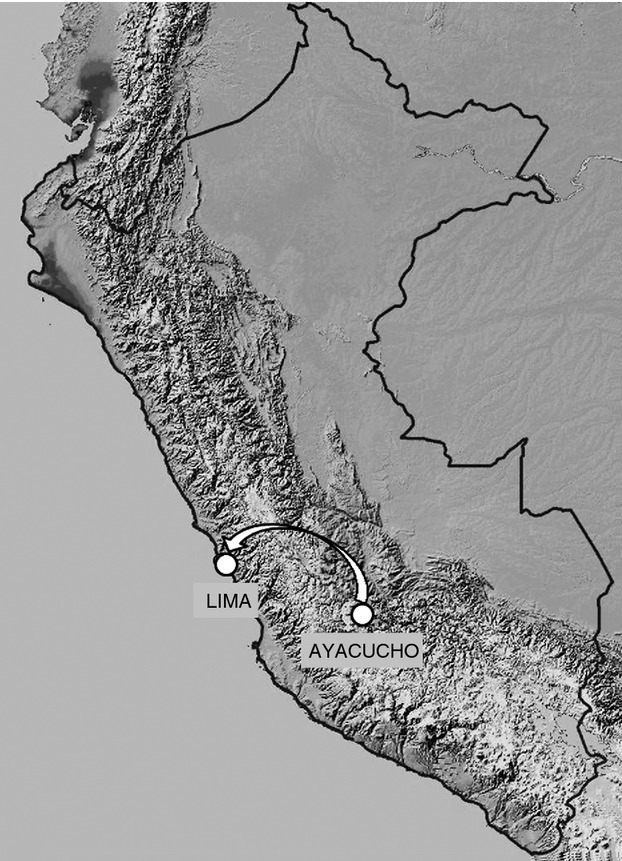
Peru map showing the locations of Secce (Ayacucho) and Lima (modified from reference 11).

The study expanded an ongoing study, reported elsewhere, that aimed to determine the relationship between migration and chronic diseases 9,10. For that parent study, the populations were San Jose de Secce, population 7215 inhabitants, a village in Ayacucho located in the Central/Southern Peruvian Highlands, Figure 1; 11 and a shanty town, Pampas de San Juan de Miraflores in Lima, population 362 643 inhabitants.

### Setting

San Jose de Secce, rural site in Ayacucho, is located in one of the most severely affected areas during the period of terrorist violence 12, where a large proportion of the population had migrated to urban areas. By the time of the study, 91% of Ayacucho's inhabitants lived in a rural area and 97% spoke Quechua, a native language 13.

Pampas de San Juan de Miraflores was the urban area for the study. It is a typical shanty town where migrants from rural areas have moved into Lima, Peru's capital, and settled over the years. Migrants from the Southern provinces, including Ayacucho, are more likely to settle in San Juan de Miraflores. Both urban and rural-to-urban migrant participants were selected from this 100% urban area. Only 11% learnt a native language during childhood and, based on place of birth, 50% were classified as migrants 13. Travel from San Jose de Secce to Lima (310 km) takes 12–15 h by bus due to the mountainous geography of the Andean region.

### Sampling

In the parent study, a single-stage random sampling method was used in all groups. For all study groups, individuals from both sexes aged 30 years and older, to allow the evaluation of the effects of time from migration, and permanently living in their residence were considered eligible. The sampling frames of adults > 30 years were 398, 1785 and 4621 for the rural (Ayacucho), rural-to-urban migrants (Ayacucho to Lima) and urban (Lima) groups, respectively. The selection of participants was stratified by 5-year age groups and sex. The original study enrolled a total of 989 people, 201 people in the rural group, 199 in the urban group and 589 migrants 9,10.

### Evaluations and exams

The parent study gathered information in different modules including a socio-demographic, migration and chronic disease's risk factor surveys, a clinical examination and laboratory tests, as reported elsewhere 9. For this study, all consenting individuals were screened for seizures using a standardised 9-question survey 14–16. A trained neurologist interviewed positive survey's respondents to confirm or rule out a diagnosis of seizures or epilepsy according to the definitions provided below.

An aliquot of 100 ul of serum was separated from archive samples collected during the parent study and processed for specific antibodies against cysticercosis on enzyme-linked immunoelectrotransfer blot (EITB, Western blot) assay using purified glycoprotein antigens 17.

### Operational definitions

*Positive survey's respondent* refers to any individual with an affirmative response to one or more of the nine questions 14–16. *Individual with seizures* refers to any individual describing one or more seizure events, provoked or unprovoked. A diagnosis of seizures does not depend on confirmatory testing, but it is based on interview data from the patient or from witnesses. A normal clinical examination or a negative EEG does not exclude the diagnosis. Seizures were typified according to the classification of seizure types of the International League Against Epilepsy 18. *Individual with epilepsy* refers to any individual describing two or more unprovoked seizures in a period of more than 24 h 19,20. *Active epilepsy* refers to an individual with epilepsy and at least one epileptic seizure in the last 5 years preceding the interview 19,20. *Inactive epilepsy* refers to an individual with a history of epilepsy but no epileptic seizures in the last 5 years preceding the interview 19,20. *Positive EITB* refers to antibody response to at least one of the seven LL-GP-purified antigens used in the assay 17. Positive EITB responses were categorised as *weak*, responses to 1 or 2 bands; *intermediate*, 3 antibody bands; or *strong*, 4–7 antibody bands.

### Data analysis

Differences in proportions were assessed using chi-square or Fisher′s exact test. Prevalences of seizures and epilepsy (per 1000 people) are presented as minimal estimates as they represent confirmed cases, even when only a proportion of survey-positive individuals attended the neurological examination for case confirmation. Cysticercosis antibody seroprevalence was calculated for each study group by age strata. In the case of migrants, seroprevalence was also calculated by time from migration. Multivariable analysis was performed using logistic regression and having epilepsy as outcome of interest. Odds ratios (OR) and 95% confidence intervals (95% CI) were calculated. All analyses were conducted using Stata v12.1 (Stata Corporation, College Station, TX, USA). Two-sided *P*-values < 0.05 were considered statistically significant.

### Ethical approval

This ancillary study and consent forms were reviewed and approved by Institutional Review Board of the Universidad Peruana Cayetano Heredia, Lima, Peru.

## Results

This study enrolled a total of 987 of the 989 participants from the parent study, 522 females (53%), mean age 48 years (range 30–93). As expected, due to the stratified sampling method, there were no differences in age or sex between the three studied populations.

### Seizure survey and neurological evaluation

From 987 participants enrolled in this study, almost all (*n* = 985) responded to the seizure survey and 278/985 were found to be positive survey respondents. Of these, 68% (188/278) were further evaluated by a neurologist. The proportions of positive respondents to the survey and the coverage of the neurological evaluation were similar between study groups, except for a slightly lower proportion of urban inhabitants attending the neurological evaluation (Table1).

**Table 1:** Studied populations, coverage of evaluations and minimal prevalence of seizures and epilepsy

Group	Urban	Migrant	Rural
N	196	589	200
Male	89 (45.4%)	280 (47.5%)	95 (42.5%)
Mean age	49.0	48.6	49.3
Age range	31–68	31–89	30–93
Positive surveys (all)	46 (23%)	165 (28%)	68 (34%)
Males	15/89 (16.9%)	78/280 (27.9%)	33/95 (34.7%)
Females	31/107 (29.0%)	87/309 (28.2%)	35/105 (33.3%)
Examined by neurologist (all)	27/46 (59%)	117/165 (71%)	44/68 (65%)
Males	7/15 (46.7%)	52/78 (66.7%)	15/33 (45.5%)
Females	20/31 (64.5%)	65/87 (74.7%)	29/35 (82.9%)
Active epilepsy	1	13	4
Inactive epilepsy	2	8	1
All epilepsies	3 (15.3/1000)	21 (35.6/1000)	5 (25.0/1000)
Single seizure	1	3	1
All seizures	4 (20.4/1000)	24 (40.7/1000)	6 (29.9/1000)

The prevalence of epilepsy (per 1000 people) was higher in migrants (35.6) than in rural inhabitants (25) and lower in the urban group (15.3), although these differences did not reach statistical significance (*P* = 0.191 and *P* = 0.160, respectively). There was no evidence of a difference in overall prevalence of epilepsy or seizures between males and females (13/464 *vs*. 21/521, *P* = 0.292). All six cases of seizures in the rural population, as well as three out of four in the urban group, were females. This led us to evaluate the proportions of positive survey respondents by sex in each study population and how many of these completed the neurological consultation for case confirmation (Table1). More positive female respondents attended the neurological consultation in the rural group (29/35, 83% *vs*. 15/33 males, 46%, OR 5.8, 95% CI 1.9–17.7, *P* = 0.001).

The age of onset of epilepsy was under 10 years in only two cases (7%), from 11 to 20 years in 8 (29%), from 20 to 30 years in 4 (14%) and from 31 to 60 years in the remaining 14 (50%). In one case, we were not able to define an age of onset of seizures.

### Serology according to migration exposure

**Figure 2:**
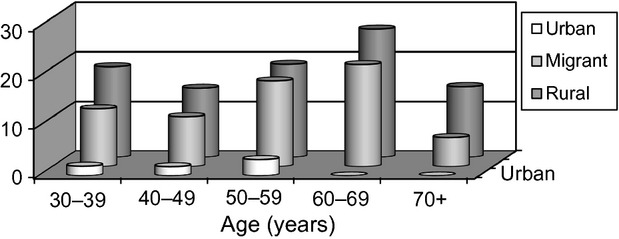
Seroprevalence of antibodies to cysticercosis by age group in rural, migrant and urban populations in Peru.

Of 985 survey respondents, 981 had an archived serum sample, all of which were processed for cysticercosis antibodies on EITB. A gradient in cysticercosis antibody seroprevalence was observed, being higher in individuals living in rural villages, less so in migrants and much lower in urban residents (Table2). The curves of seroprevalence by age demonstrated parallel trends between individuals living in rural villages and migrants, with a much lower seroprevalence in the urban group (Figure 2).

**Table 2:** Seroprevalence and number of reactive bands on EITB by population origin

Group	Urban	Migrant	Rural
N	195	587	199
Seropositive	4 (2.1%)	79 (13.5%)	36 (18.1%)
1–2 bands	3 (1.5%)	32 (5.5%)	8 (4.0%)
3 bands	1 (0.5%)	45 (7.7%)	23 (11.6%)
4–7 bands	0 (0%)	2 (0.3%)	5 (2.5%)
Seroprevalence in individuals with seizures	0/4 (0%)	3/24 (12.5%)	4/6 (66.7%)
Seroprevalence in negative survey respondents	1/149 (0.7%)	55/423 (13.0%)	20/131 (15.3%)

**Figure 3:**
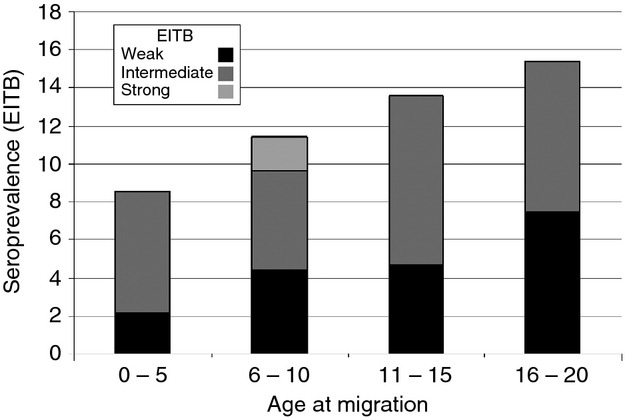
Current serum antibody responses by age at migration in individuals who moved (approximately 30 years ago) from an endemic rural region into a non-endemic city.

Intragroup analysis of the migrant population showed that seroprevalence consistently increased according to the age that the individual had at the time of leaving their rural villages, between age 0 and 15 years or older. These findings suggest cumulative exposure while in their rural villages: the older the age at migration from the rural area, the higher the seroprevalence of antibodies (Figure 3).

Given that the study participants migrated a long time ago, we also assessed the effect of the time elapsed since migration. Seroprevalence in the migrant group decreased with time away from their native villages (16.5% [46/279] in the initial 25 years, 12.2% [25/205] between 25 and 40 years, and 8.0% [8/103] in those away for more than 40 years), suggesting that antibodies wane over a long time in a sizable proportion of seropositive individuals. Adjusting by time away from the rural area did not affect the increasing pattern of serology by age at migration.

### Relation between seroprevalence and seizures

In rural villagers, there was strong evidence of an association between positive EITB and having seizures (4/6 *vs*. 32/193, *P* = 0.011, Fisher's exact test). This association was not observed in long-term migrants or in urban residents.

Of seven individuals reacting to four or more specific antibody bands on EITB, three had active epilepsy (two of five in rural group and one of two migrants). There was evidence of an association between strong response (≥ 4 antibody bands) on EITB with seizures (3/29 *vs*. 4/952, *P* < 0.001, Fisher's exact test). Logistic regression analysis for epilepsy confirmed this observation: compared with seronegative participants, those with a strong positive serology response were 12 times more likely to have epilepsy, after adjusting by study group (urban, migrant and rural) (Table3).

**Table 3:** Factors associated with epilepsy in urban, migrant and rural individuals in Peru (*n* = 985)

	Odds ratio	95% C.I.	*P*-value
Endemicity			
No (urban)	Reference		
Endemic (migrant, rural)	2.09	0.62–7.08	0.235
Serology response			
Seronegative	Reference		
1 or 2 antibody bands	0.80	0.10–6.06	0.826
3 antibody bands	0.97	0.22–4.23	0.966
4 or more antibody bands	12.79	3.16–51.62	**< 0.001**

Bold value indicate significant results.

## Discussion

We found strong evidence of differences in antibody profiles and weaker evidence of higher seizure prevalence in rural villagers and long-term migrants compared with residents of a non-endemic urban shanty town in Lima, Peru. The prevalence of serum antibodies was higher in rural habitants and moderately high in migrants but much lower in the urban population. In the context where most studies of cysticercosis from non-endemic areas focus on international migration as a source of transmission – we were unable to find any publication related to changes in cysticercosis prevalence related to within-country migration – this report demonstrates that within-country rural-to-urban migration also carries a considerable burden, in terms of symptoms and immunological responses that last over time. As such our results confirm the impact of the disease in rural areas and contribute to defining high-risk populations in apparently non-endemic settings.

In rural villagers, there was a significant association between antibody seropositivity and seizures, yet this association did not hold in long-term migrants. This most likely reflects the chronic nature of seizures in relation to waning of antibodies along the years in a sizable proportion of individuals with NCC – those who resolve the infection with or without residual brain calcifications. However, a significant association between epilepsy and having a strong serological reaction, ≥ 4 antibody bands, was found in the overall study population and persisted after adjusting by population of origin – suggesting that epilepsy is present in individuals with heavier or more severe cysticercosis infections.

Seizures were more frequent in individuals with a strong positive serological response and in people born in a rural endemic region, both in rural residents and in long-term migrants. The unexpected finding of all six cases of seizures in rural villagers being female seems associated to male positive survey respondents failing to attend the neurological consultation for case definition, suggesting a subdiagnosis of seizure cases in male rural villagers due to shame or fear to stigma. Also, the data on age of onset of seizures show a very low proportion of seizure disorders beginning in childhood. This is most likely affected by recall bias as our population was evaluated many years after (all participants were 30 years or older). The sample size, calculated for the parent study and powered to yield differences on cardiovascular outcomes, did not allow confirming the increased odds ratios for seizures (1.9) and epilepsy (2.2) found in this study for the rural, rural-resident and rural-to-urban migrant, population groups.

Another limitation of this study is the lack of brain imaging studies. Computed tomography (CT) scans were not feasible due to the rural nature of San Jose de Secce, without access to a CT scanner or even less to a MRI machine, which is a typical situation for heavily endemic rural regions. This limitation also impeded to assess the presence or frequency of individuals with acute symptomatic seizures. Chances for selection bias are less likely. Because migration is often driven by economic and other factors that are likely to be related to health, migrants rarely are representative of the rural area they come from. Terrorism and political violence led to mass rural-to-urban migration in Peru. A large proportion of the population migrated because of the need to escape from violence rather than effects of economic forces or need for health care. Thus, the selection bias among migrants was reduced 21.

Both, rural residents and rural-to-urban migrants had a much higher frequency of antibodies to cysticercosis, with stronger reactions in rural villagers, seemingly the strongest associated factor to seizures among those studied here. The association between seizures and serology disappears in the migrant population, yet the overall seizure and epilepsy prevalence were still high in this group. In addition to this, a pattern towards a higher seroprevalence of serum antibodies was observed among rural-to-urban migrants by age at migration: the higher the age at migration from the rural area, the higher the seroprevalence of antibodies.

The comparisons between rural, migrant and urban populations presented in this study confirm the association between cysticercosis and seizures in rural endemic communities, expose clear differences in endemicity within an endemic country and suggest that the neurological consequences of cysticercosis infection likely outlast the antibody response for years.
